# IGFL2-AS1, a Long Non-Coding RNA, Is Associated with Radioresistance in Colorectal Cancer

**DOI:** 10.3390/ijms24020978

**Published:** 2023-01-04

**Authors:** Jeeyong Lee, Da Yeon Kim, Younjoo Kim, Ui Sup Shin, Kwang Seok Kim, Eun Ju Kim

**Affiliations:** 1 Division of Radiation Biomedical Research, Korea Institute of Radiological and Medical Sciences, Seoul 01812, Republic of Korea; 2 Department of Radiological and Medico-Oncological Sciences, University of Science and Technology, Daejeon 34113, Republic of Korea; 3 Department of Radiological and Clinical Research, Korea Cancer Center Hospital, Korea Institute of Radiological and Medical Sciences, Seoul 01812, Republic of Korea; 4 Department of Internal Medicine, Korea Cancer Center Hospital, Korea Institute of Radiological and Medical Sciences, Seoul 01812, Republic of Korea; 5 Department of Surgery, Korea Cancer Center Hospital, Korea Institute of Radiological and Medical Sciences, Seoul 01812, Republic of Korea

**Keywords:** IGFL2-AS1, long non-coding RNA, radioresistance, AKT, biomarker

## Abstract

Precise prediction of radioresistance is an important factor in the treatment of colorectal cancer (CRC). To discover genes that regulate the radioresistance of CRCs, we analyzed an RNA sequencing dataset of patient-originated samples. Among various candidates, IGFL2-AS1, a long non-coding RNA (lncRNA), exhibited an expression pattern that was well correlated with radioresistance. IGFL2-AS1 is known to be highly expressed in various cancers and functions as a competing endogenous RNA. To further investigate the role of IGFL2-AS1 in radioresistance, which has not yet been studied, we assessed the amount of IGFL2-AS1 transcripts in CRC cell lines with varying degrees of radioresistance. This analysis showed that the more radioresistant the cell line, the higher the level of IGFL2-AS1 transcripts—a similar trend was observed in CRC samples. To directly assess the relationship between IGFL2-AS1 and radioresistance, we generated a CRC cell line stably expressing a small hairpin RNA (shRNA) targeting IGFL2-AS1. shRNA-mediated knockdown of IGFL2-AS1 decreased radioresistance and cell migration in vitro, establishing a functional role for IGFL2-AS1 in radioresistance. We also showed that downstream effectors of the AKT pathway played crucial roles. These data suggest that IGFL2-AS1 contributes to the acquisition of radioresistance by regulating the AKT pathway.

## 1. Introduction

Colorectal cancer (CRC) ranks high in mortality among cancers because it tends to be symptomless until quite advanced [1]. There are treatment options for CRCs that depend on the genetic heterogeneity, cancer stage, and spatial distribution, but a prompt and proper choice of treatment is crucial. Among many factors, radioresistance is a major cause of treatment failure. Therefore, precise prediction of radioresistance is important for the successful outcome of a patient’s specific treatment.

A previous study analyzed the profiles of CRC patient tumors according to their radioresistance [2]. In that study, biological and bioinformatic methods were used to analyze the candidate proteins that contribute to radioresistance, but biomolecules other than proteins were omitted from further analysis. However, the vast majority of RNA transcripts do not encode proteins, and many still display biological functions [3]. Such non-coding RNAs are emerging as new functional participants in various physiological processes.

In the current study, a detailed re-analysis of the previous RNA sequencing (RNA-seq) dataset revealed non-coding RNAs among differentially transcribed genes. Of these, IGFL2-AS1, a long non-coding RNA (lncRNA), drew our attention. LncRNAs are a class of non-protein-coding RNAs longer than 200 nucleotides that possess biological functions in transcriptional silencing, transcriptional activation, chromosomal modification, and intranuclear transport [4]. To date, a handful of studies performed on IGFL2-AS1 have reported higher levels of this transcript in renal cell carcinoma [5], cervical cancer [6], oral cancer [7], and gastric cancer [8]. IGFL2-AS1 appears to play a role in promoting cancer that involves effects on cell proliferation, migration, and epithelial-mesenchymal transition (EMT). Among the pathways reported to be regulated by IGFL2-AS1 are Wnt/β-catenin signaling via the homeobox protein SATB1 [7] and growth factor signaling via the serine/threonine phosphatase PP2A [8]. Ma et al. reported that PP2A controls AKT activation, which in turn regulates cell proliferation and migration [8].

Although previous studies have argued that IGFL2-AS1 could regulate cancer development, the role of IGFL2-AS1 in the biological regulation of radioresistance has not been studied. Accordingly, we hypothesized that, in addition to contributing to cancer progression, IGFL2-AS1 transcripts play a role in the acquisition of radioresistance. To test this hypothesis, we investigated IGFL2-AS1 transcript levels in CRC cell lines as a function of the degree of radioresistance in each cell line. Focusing on radioresistance, proliferation, and migration, which could significantly affect cancer prognosis, we also investigated the biological effects of silencing IGFL2-AS1 by generating a CRC cell line in which IGFL2-AS1 was stably knocked down using an RNA interference approach employing short hairpin RNA (shRNA). Collectively, the data presented here, including the identification of IGFL2-AS1 as a candidate target, could improve the application and effectiveness of radiation therapy and aid in the discovery of new drugs for treating CRC.

## 2. Results

### 2.1. Identification of IGFL2-AS1 as a Candidate Regulator of Radioresistance in CRC

**Figure 1:**
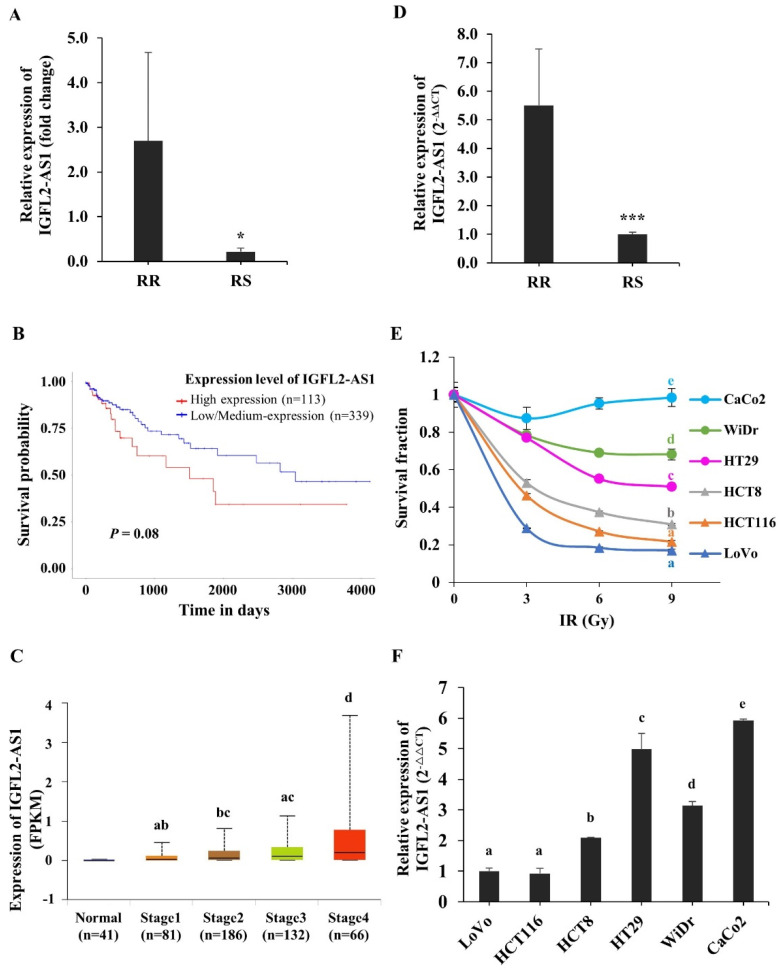
Identification of IGFL2-AS1 as a candidate regulator of radioresistance in CRC. (**A**) IGFL2-AS1 levels in a CRC patient RNA-seq dataset. The graph depicts the degree of differential expression of IGFL2-AS1 transcripts between radioresistant (RR) and radiosensitive (RS) patient-originated samples. (**B**) Survival analysis of IGFL2-AS1 expression in colon adenocarcinoma. The results based on the UALCAN database indicated that higher expression of IGFL2-AS1 was associated with poor prognosis in colon adenocarcinoma (*p* = 0.08). (**C**) Relationship between IGFL2-AS1 expression and individual cancer stages of patients with colon adenocarcinoma in UALCAN database. (**D**) The relative level of IGFL2-AS1 transcripts in the patient-originated samples, measured using qRT-PCR. (**E**) Viability of CRC cell lines after irradiation with doses of 0, 3, 6, and 9 Gy (*n* = 3 independent experiments), measured by MTT assay. Survival fractions were assessed 3 days after irradiation. Data are normalized to those of non-irradiated controls. (**F**) The relative level of IGFL2-AS1 transcripts, measured using qRT-PCR. The graph depicts the degree of differential expression of IGFL2-AS1 transcripts between CRC cell lines. Data are normalized to those of the LoVo cell line. Data are presented as means ± SD. Student’s *t-*test (**C**) and one-way ANOVA with Tukey post-hoc tests (**E**,**F**) were performed. Different letters on top represent statistically significant results (*p* < 0.05), whereas the same letters indicate no statistically significant differences. Asterisk indicates the followings: * *p* < 0.05, *** *p* < 0.001.

To investigate genes involved in regulating radioresistance in CRC, we analyzed RNA-seq data from patient-originated samples. The clinical features of patients are listed in Supplementary Table S1. Radioresistant (RR) and radiosensitive (RS) patients were defined according to the pathological stage of their surgical specimens after neoadjuvant chemoradiation treatment. To identify genes associated with radioresistance, we specifically focused on differentially transcribed non-coding RNAs in RR and RS patient-originated samples. Among genes with large fold changes, the lncRNA, IGFL2-AS1, was selected (Figure 1A and Supplementary Table S1). The relative levels of IGFL2-AS1 transcripts were higher in RR patient samples, suggesting a possible role for IGFL2-AS1 in radioresistance. Interestingly, higher levels of IGFL2-AS1 transcript have been reported in various cancers [5,6,7,8], and an esophageal cancer-trap project [9] in cBioPortal [10] showed that the IGFL2-AS1 gene was amplified in cancer (Supplementary Figure S1). High levels of IGFL2-AS1 transcripts were also associated with poor disease-free survival (Figure 1B, Supplementary Figure S2, and Table 1) and advanced cancer stages (Figure 1C). These data suggest a possible role for IGFL2-AS1 in cancer progression. A quantitative real-time PCR (qRT-PCR) analysis of IGFL2-AS1 transcript levels showed that transcripts were ~5-fold more abundant in RR patients’ samples (Figure 1D), strengthening the validity of RNA-seq analyses.

To investigate the relationship between radioresistance and IGFL2-AS1, we employed well-known CRC cell lines, first testing the radioresistance of the respective cell lines (Figure 1E and Supplementary Figure S3). The rank order of radioresistance of CRC cell lines from low to high based on MTT assays was LoVo, HCT116, HCT8, HT29, WiDr, and CaCo2. We then examined the level of IGFL2-AS1 transcripts in the respective cell lines. As shown in Figure 1F, the more radioresistant the cell line, the higher the level of IGFL2-AS1 transcripts. Overall, these data suggest that IGFL2-AS1 is a good candidate regulator of radioresistance and indicate that the level of IGFL2-AS1 transcript could be used as a biomarker for discriminating the radioresistance of CRC.

### 2.2. Generation and Characterization of a CRC Cell Line Stably Transfected with sh-IGFL2-AS1

**Figure 2:**
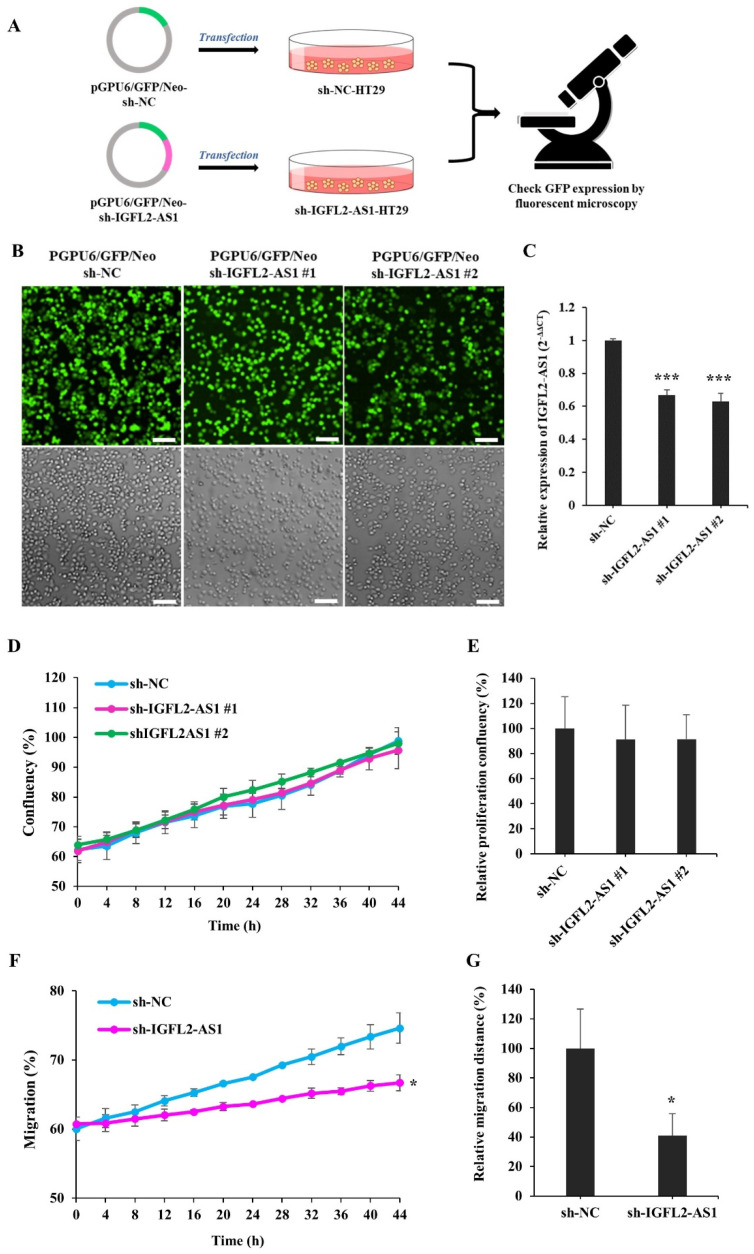
Generation and characterization of a CRC cell line stably transfected with sh-IGFL2-AS1. (**A**) Schematic depicting the establishment of stable cell lines. HT29 cells were transfected with the shRNA-expressing plasmids, pGPU6/GFP/Neo-sh-NC (sh-NC) and pGPU6/GFP/Neo-sh-IGFL2- AS1 (sh-IGFL2-AS1). After incubating transfected HT29 cells with G418 for 2 weeks, individual colonies were isolated. (**B**) GFP fluorescence (upper panels) and brightfield (lower panels) images of the indicated cell lines are shown. Scale bar: 400 μm. (**C**) The relative level of IGFL2-AS1 transcripts, measured using qRT-PCR. The graph depicts IGFL2-AS1 transcript levels in the indicated cell lines. Data are normalized to those of the sh-NC-HT29 cell line. (**D**) Proliferation rates of the indicated cell lines, measured using a Celloger Mini Plus time-lapse microscopy system. The graph shows proliferation rates calculated from digital images acquired from the same field over 48 h. (**E**) Graph showing the proliferation rates of the indicated cell lines after monitoring for 48 h. (**F**) Wound-healing assays performed using the indicated cell lines. The scratched area was monitored using a time-lapse microscope. The graph shows the rate of cell migration measured over a period of 48 h. (**G**) Graph showing wound-healing assays for the indicated cell lines, monitored after 48 h. Data are presented as means ± SD. Asterisk indicates the followings: * *p* < 0.05, *** *p* < 0.001.

The role of IGFL2-AS1 in radioresistance has not been examined. Since IGFL2-AS1 transcript levels were high in radioresistant cells (Figure 1), we tested the possibility that a reduction of IGFL2-AS1 transcript would decrease radioresistance. To this end, we generated an sh-IGFL2-AS1 plasmid targeting IGFL2-AS1 (pGPU6/GFP/Neo-sh-IGFL2- AS1) and a control, non-targeting sh-NC plasmid (pGPU6/GFP/Neo-sh-NC), as depicted in Figure 2A. We further selected HT29 cells for transfection because these cells exhibited moderate radioresistance (Figure 1E), making it possible to readily assess modulation in either direction by the shRNA plasmid. Because the efficiency of transient transfection was low, we then stably transfected these cells with shRNA plasmid (Figure 2A; for details, see Materials and methods), which is advantageous because all cells exhibiting the desired gene knockdown can be identified and maintained. HT29 cells transfected with sh-IGFL2-AS1 or sh-NC were incubated with G418 for 2 weeks and then individual colonies were picked. The resulting stable clones were G418 resistant and positive for GFP (Figure 2B). Notably, IGFL2-AS1 transcript levels were significantly decreased in sh-IGFL2-AS1-HT29 cell lines compared with the sh-NC-HT29 cell line (Figure 2C).

Cen et al. previously reported that siRNA targeting IGFL2-AS1 inhibited cell growth [11]. Because the reported reduction in proliferation could complicate the subsequent interpretation of data, we assessed the proliferation rate of the sh-IGFL2-AS1-HT29 cell line relative to that of the sh-NC-HT29 cell line. Proliferation was assessed in two sh-IGFL2-AS1-HT29 clones, and digital images were acquired from the same field over a 48-h period by time-lapse microscopy using a Celloger Mini Plus system (see Materials and Methods). The resulting images were processed and the degree of confluence was measured. An analysis of the proliferation of sh-IGFL2-AS1-HT29 cell lines showed no significant difference compared with the sh-NC-HT29 cell line (Figure 2D,E and Supplementary Figure S4). Thus, we conclude that sh-IGFL2-AS1 minimally affects cell proliferation, at least in our experimental conditions.

Cen et al. also reported that the reduction of IGFL2-AS1 inhibited cell migration [11]. To validate this finding, we assessed the migration of sh-IGFL2-AS1-HT29 using wound-healing assays, in which the rate of cell migration into a scratched area in a monolayer mimicking an incision wound was monitored over a 48-h period (Figure 2F,G and Supplementary Figure S5). shRNA-mediated IGFL2-AS1 knockdown decreased the rate of HT29 cell migration compared with control cells, confirming that IGFL2-AS1 is involved in the cell migration process. Taken together, these data confirm successful stable transfection of sh-IGFL2-AS1-HT29 cells and demonstrate that a reduction in IGFL2-AS1 transcript levels inhibits cell migration but not cell proliferation.

### 2.3. Radiation Sensitivity Is Enhanced in IGFL2-AS1–Knockdown CRC Cell Lines

**Figure 3:**
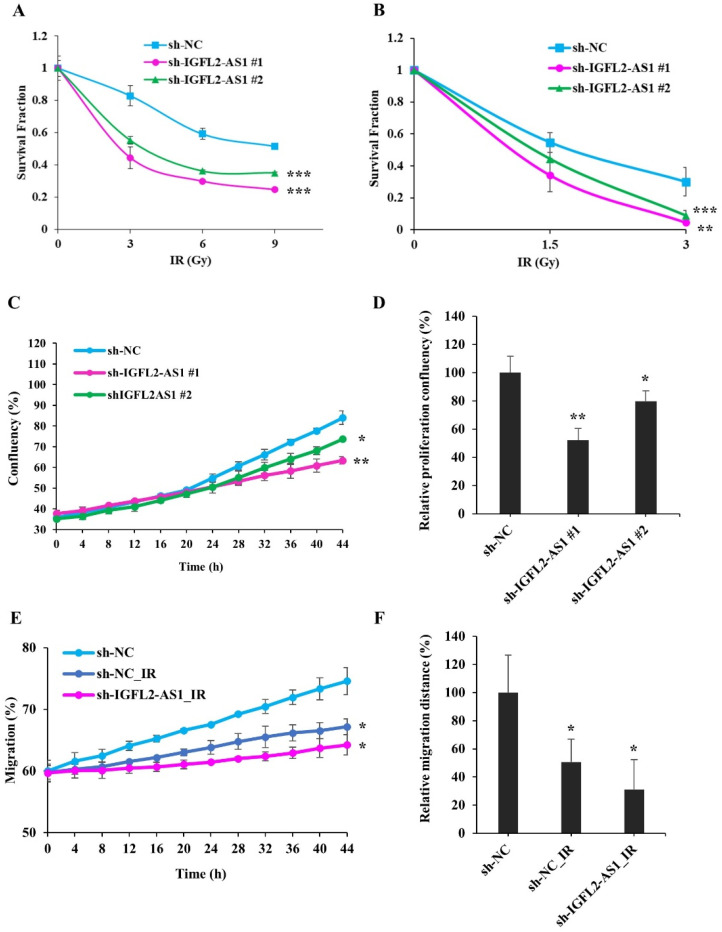
Radiation sensitivity is enhanced in IGFL2-AS1–knockdown CRC cell lines. (**A**) Viability of the indicated cell lines after irradiating with doses of 0, 3, 6, and 9 Gy, measured by MTT assay. Survival fractions were assessed 3 days after irradiation. Data are normalized to those of non-irradiated controls. (**B**) Colony formations after irradiating with doses of 0, 1.5, 3, Gy, measured by clonogenic assay. Colonies containing more than 50 cells were assessed 14 days after irradiation. Data were normalized to those of non-irradiated controls and the results are expressed as surviving fraction. (**C**) Proliferation rates of the indicated cell lines following exposure to 3 Gy irradiation (at 0 h). Digital images from the same field were acquired using a Celloger Mini Plus time-lapse microscopy system. The graph shows proliferation rates calculated over a 48-h period. (**D**) Graph showing proliferation rates of the indicated cell lines over a 48-h period after irradiation at a dose of 3 Gy (at 0 h). (**E**) Wound-healing assays performed using the indicated cell lines. Cells were irradiated at a dose of 3 Gy irradiation (at 0 h), and the scratched area was subsequently monitored for migrated cells using time-lapse microscopy. (**F**) Graph showing the rate of cell migration, measured over a period of 48 h after wounding and/or irradiating (at 0 h). Data are presented as means ± SD. Asterisk indicates the followings: * *p* < 0.05, ** *p* < 0.01, *** *p* < 0.001.

To investigate the relationship between radioresistance and IGFL2-AS1, we further examined the characteristics of sh-IGFL2-AS1-HT29 cell lines, first assessing their radioresistance by MTT assay (Figure 3A) and clonogenic assay (Figure 3B). These assays indicated a significant reduction in radioresistance in sh-IGFL2-AS1-HT29 cell lines compared with the control sh-NC-HT29 cell line.

Next, we measured the proliferation of sh-IGFL2-AS1-HT29 cell lines following irradiation using the Celloger Mini Plus time-lapse microscopy system. Cells were γ-irradiated with 3 Gy at 0 h, and cell confluence was measured from the same field every 2 h over a period of 48 h (Figure 3C,D and Supplementary Figure S6). Interestingly, graphs of the proliferation of the hNC-HT29 cell line showed a biphasic slope, with the proliferation rate accelerating after 20 h, suggesting that these control cells recover from irradiation damage at this point and resume their normal proliferation rate thereafter. In contrast, sh-IGFL2-AS1-HT29 cell lines showed no biphasic proliferation curve and continued to proliferate at the same rate, indicating that the recovery process observed in control cells was compromised in the shAS1-HT29 cell line.

Because IGFL2-AS1 is associated with cell migration (Figure 2F,G and Supplementary Figure S5), we investigated the effect of irradiation on cell migration (Figure 3E,F and Supplementary Figure S7). We found that migration was compromised in the sh-NC-HT29 cell line. Notably, irradiation combined with sh-IGFL2-AS1 further attenuated cell migration.

### 2.4. AKT Pathway Signaling Is Suppressed by sh-IGFL2-AS1

**Figure 4:**
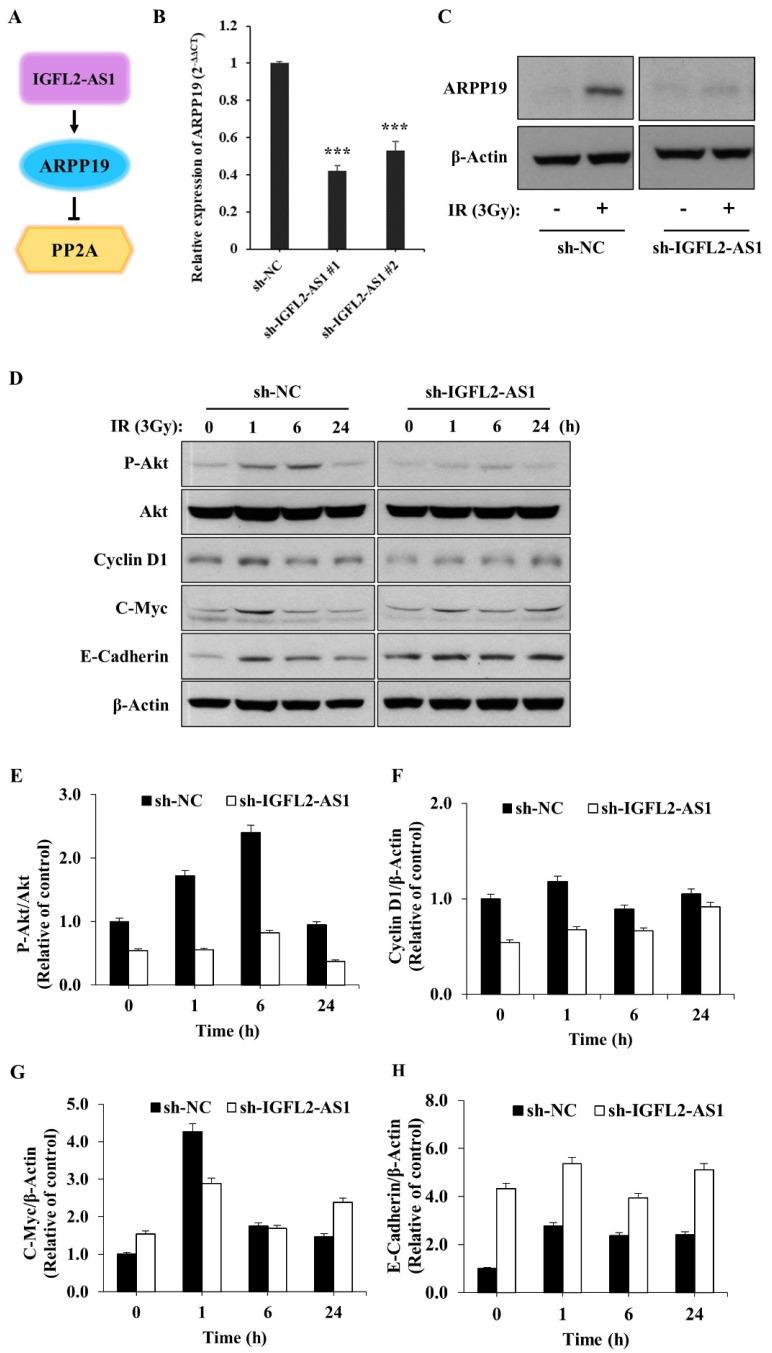
AKT pathway signaling is suppressed by sh-IGFL2-AS1. (**A**) Schematic depicting the IGFL2-AS1 pathway. (**B**) Relative transcription of ARPP19, measured using qRT PCR. The graph depicts the levels of ARPP19 in the indicated cell lines. Data are normalized to those of the sh-NC-HT29 cell line; error bars indicate SDs. *** *p* < 0.001. (**C**) Protein expression of ARPP19, measured by western blotting. The expression of β-actin was used as a loading control. (**D**) Western blot analyses of various proteins downstream of AKT. Cells incubated as indicated after irradiation (3 Gy) were lysed and analyzed by western blotting; β-actin was used as a loading control. (**E**–**H**) Densitometric measurements of protein expressions, corresponding to (**D**). The results are presented as the ratio of phosphor-AKT/AKT (**E**), cyclin D1/β-actin (**F**), c-Myc/β-actin (**G**), and E-cadherin/β-actin (**H**).

The lncRNA, IGFL2-AS1, could act as a competing endogenous RNA to regulate various transcriptional processes, as depicted in Figure 4A. In this context, Ma et al. previously reported that IGFL2-AS1 was capable of enhancing the level of ARPP19 (cAMP regulated phosphoprotein 19) [8]. To confirm this report and provide insight into the underlying mechanism of IGFL2-AS1 in radioresistance, ARPP19 levels were measured in the CRC cell lines (Supplementary Figure S8). The data showed the strong correlation between ARPP19 level and radioresistance. Next, we also quantified ARPP19 levels in the presence of sh-IGFL2-AS1 using qRT-PCR (Figure 4B) and western blot (Figure 4C). These analyses showed that ARPP19 levels were significantly downregulated in sh-IGFL2-AS1-HT29 cells compared with control cells, as expected. Interestingly, the level of ARPP19 was greatly upregulated upon irradiation, suggesting a possible role in radioresistance. Because ARPP19 is a protein phosphatase 2A (PP2A) inhibitor and can activate AKT, we assessed AKT activation in sh-IGFL2-AS1-HT29 and sh-NC-HT29 cells by monitoring levels of the phosphorylated (active) form of AKT [12]. Compared with control sh-NC-HT29 cells, irradiation-induced AKT phosphorylation was dramatically reduced in sh-IGFL2-AS1-HT29 cells (Figure 4D,E), suggesting that knockdown of IGFL2-AS1 transcripts enhances the activity of PP2A phosphatase, which in turn dephosphorylates and inactivates AKT.

Upregulation of the AKT pathway has been linked to radiotherapy resistance [13]. Because our data suggest that IGFL2-AS1 controls AKT activation, we tested the involvement of various signaling pathways known to act downstream of AKT by western blot analysis (Figure 4D–H). First, we checked the proteins involved in promoting cell growth. This analysis showed that the levels of cyclin D1, a cell cycle controller [14], and c-Myc, a cellular proto-oncogene [15], were decreased in sh-IGFL2-AS1-HT29 cells compared with controls. We further found that, in non-irradiated cells, the levels of E-cadherin, which is involved in controlling cell migration [16], were much higher in sh-IGFL2-AS1-HT29 cells compared with control cells. These data could explain the observed compromised cell migration in the absence of irradiation in sh-IGFL2-AS1 cells (Figure 2F,G and Supplementary Figure S5). Taken together, these observations suggest that sh-IGFL2-AS1 suppresses AKT signaling and its downstream consequences, including cell proliferation and cell migration.

## 3. Discussion

Achieving efficient and adequate control of CRC requires prompt selection of the appropriate treatments. Since the development of radioresistance constitutes a major cause of treatment failure, the prediction of radioresistance is important for success of a patient’s specific treatment. In this report, we analyzed non-coding RNAs—new functional participants in various physiological processes—by RNA-seq analysis of a CRC patient dataset. This next-generation sequencing technology has made it possible to identify specific expression patterns of non-coding RNAs.

The potential of lncRNAs as novel biomarkers and therapeutic targets has been growing [17,18,19]. Various lncRNAs have been suggested to be involved in CRC development and possibly affect the chemo- and radioresistance of CRC [20,21]. It has been shown that some lncRNAs, including lncRNA-p21 [22] and OIP5-AS1 [23], are downregulated in CRC and enhance the radiation sensitivity of CRC cells when overexpressed. In contrast, the knockdown of other lncRNAs, such as MALAT1 [24] and HOTAIR [25], has been shown to enhance radiation sensitivity.

Our analysis revealed IGFL2-AS1 as a differentially transcribed gene that is highly expressed in radioresistant CRC compared with radiosensitive CRC. Recent research has revealed that IGFL2-AS1 is upregulated in a wide variety of human cancers, where it participates in cancer progression, and that it exerts complex functions depending on the type of cancer [5,7,8,11,26]. A recent study found that IGFL2-AS1 promotes the proliferation, migration, and invasion of CRC cells in vitro [11]. However, the biological regulation of radioresistance through IGFL2-AS1 in CRC has remained unclear.

**Figure 5:**
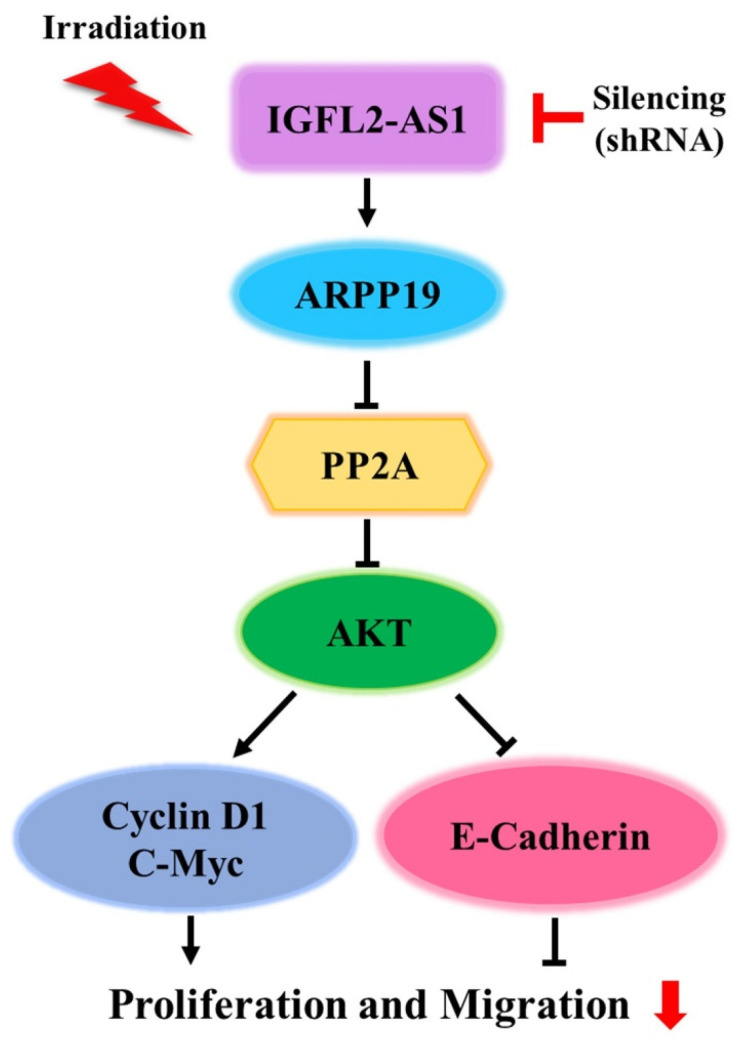
Schematic depiction of the sh-IGFL2-AS1 regulatory pathway. The current model of the association of IGFL2-AS1 with the acquisition of radioresistance in CRC.

These previous observations informed our speculation that IGFL2-AS1 might play an active biological role in the radioresistance of CRC. We confirmed that high levels of IGFL2-AS1 transcripts were positively correlated with clinicopathological features and showed that knockdown of IGFL2-AS1 sensitized CRC cells to radiation and retarded their migrations. Our research also identified a novel pathway involved in coordinating radioresistance. Specifically, we found evidence supporting a role for the AKT pathway in mediating the radioresistance-regulatory function of IGFL2-AS1. IGFL2-AS1 is known to regulate the expression of ARPP-19, a PP2A inhibitory protein [8] that has been associated with several types of human cancer [27]. PP2A is a critical negative regulator of tumorigenesis that is inhibited by endogenous inhibitory proteins in several pathological conditions, including cancer [27,28]. Importantly, PP2A profoundly affects the activity of AKT [8,28]—the main signaling pathway in radioresistance acquisition [13]—supporting our experimental findings. Therefore, we propose that sh-IGFL2-AS1 is a regulator of radioresistance acquisition that acts through the modulation of the AKT pathway. We further show that sh-RNA-induced AKT downregulation also affects cell proliferation and cell migration via cyclin D1, c-Myc, and E-cadherin proteins (Figure 5).

Various cancers differ in the molecular details by which the activity of the AKT pathway—the key signaling pathway in radioresistance—is enhanced. Therefore, many researchers have sought to develop AKT pathway inhibitors as a strategy for controlling radioresistance. However, the inherent toxicity associated with shutting down AKT activation has prevented the successful development of such radiosensitizers [13]. We showed that IGFL2-AS1 is new mechanism responsible for activating AKT signaling and conferring subsequent radioresistance. We believe that shRNA-based inhibition of AKT signaling could be a new approach for clinical applications. We would also note the potential of gene chip applications for measuring IGFL2-AS1 levels. Ultimately, our goal is to provide prognostic and diagnostic biomarkers and to improve the application and effectiveness of radiation therapy.

## 4. Materials and Methods

### 4.1. CRC Sample Preparation

Samples from six pathologically proven CRC patients were included in the study. Outcomes of neoadjuvant chemoradiation therapy (NCRT) were categorized as complete response (*n* = 3) or poor response (*n* = 3) based on an analysis of total mesorectal excision specimens. The pathologic response was evaluated using the tumor regression grade (TRG) system suggested by the Gastrointestinal Pathology Study Group of the Korean Society of Pathologists [29]. The respective samples were collected from endoscopic cancers prior to neoadjuvant chemoradiation therapy and were subsequently processed as described previously [30]. 

### 4.2. RNA-Seq and Data Analysis

Total RNA was isolated using QIAzol reagent (Qiagen, Hilden, Germany) according to the manufacturer’s instructions. RNA-seq was performed on high-quality RNA samples from cancer tissues (RNA integrity number > 7), evaluated using an Agilent 2100 Bioanalyzer. The samples were multiplexed into each lane and sequenced on a HiSeq 4000 system (Illumina, San Diego, CA, USA). The sequenced libraries were aligned to the human genome reference sequence (hg19) using HISAT v2.1.0 [31]. The reference genome sequence and its annotation were downloaded from the UCSC genome browser (https://genome.ucsc.edu/, accessed on 15 August 2020). QuantSeq 3′ mRNA-Seq reads were aligned using Bowtie2 [32]. For alignment to the genome and transcriptome, Bowtie2 indices were generated from the genome assembly sequence or the representative transcript sequences. The alignment file was used for assembling transcripts, estimating their abundances, and detecting differentially expressed genes (DEGs), the latter of which were determined based on counts from unique and multiple alignments using coverage in Bedtools [33]. Read count data were processed based on a quantile normalization method using edgeR in the Bioconductor software package [34]. Data mining and graphic visualization were performed using Excel-based Differentially Expressed Gene Analysis (ExDEGA; Ebiogen Inc., Seoul, Republic of Korea). Probe sets without corresponding gene symbols were removed. In this study, a *p*-value < 0.05 and absolute log_2_ (fold change) ≥ 1 were considered statistically significant.

### 4.3. Analyses Using Kaplan-Meier Plotter and UALCAN Databases

The prognostic significance of the mRNA expression levels of IGFL2-AS1 in various types of cancer was evaluated using a Kaplan–Meier plotter (https://kmplot.com/analysis/, accessed on 30 August 2022) and UALCAN (http://ualcan.path.uab.edu/, accessed on 30 August 2022). The two databases of Kaplan-Meier plotter [35] and UALCAN [36] contain the same RNA-seq data (from TCGA) for 21 and 35 types and subtypes of cancer, respectively. Kaplan-Meier plotter also contains databases including GEO and EGA. The overall survival rate (OS) in patients with various types and subtypes of carcinomas was estimated by Kaplan-Meier plotter databases. The prognostic significance of the mRNA expression levels of IGFL2-AS1 in colon adenocarcinoma was evaluated by UALCAN databases. A *p*-value of <0.05 was considered statistically significant.

### 4.4. Quantitative Real-Time Polymerase Chain Reaction (qRT-PCR)

Total RNA was extracted using QIAzol reagent (Qiagen) and reverse transcribed to cDNA with amfiRivert reverse transcriptase (GenDEPOT, Katy, TX, USA) according to the manufacturer’s instructions. cDNA was amplified by qRT-PCR performed on a Mic Real-Time PCR system (Bio Molecular Systems, Upper Coomera, QLD, Australia) using Luna Universal qPCR master mix (New England Biolabs Inc., Beverly, MA, USA) and the following primer pairs: IGFL2-AS1, 5′-TTG GAG GGT GAG AGA CCA CA-3′ (forward) and 5′-TGC TGC AGA ATC AAC GAC CT-3′ (reverse); ARPP19, 5′-CAA AAG CCT GGA GGT TCA GAT TTC-3′ (forward) and 5′-GTC ACC AGT GAC CTC CGT CTT A-3′ (reverse); and GAPDH, 5′-AAG GAC TCA TGA CCA CAG TC-3′ (forward) and 5′-TTC AGC TCA GGG ATG ACC TT-3′ (reverse). Results were analyzed using the ΔCT and 2^−ΔΔCT^ quantification methods.

### 4.5. Cell Lines and Maintenance

Six different CRC cell lines (LoVo, HCT116, HCT8, HT29, WiDr, and CaCo2) were obtained from the American Type Culture Collection (ATCC, Manassas, VA, USA). LoVo, HCT116, HCT8, and HT29 cells were cultured in Roswell Park Memorial Institute (RPMI-1640) medium supplemented with 10% fetal bovine serum (FBS) and 1% penicillin-streptomycin. WiDr, and CaCo2 cells were cultured in Dulbecco’s modified Eagle medium (DMEM) supplemented with 10% FBS and 1% penicillin-streptomycin. All cell lines were maintained at 37 °C in a humidified 5% CO_2_ atmosphere.

### 4.6. Transfection and Stable Cell Lines

The shRNA plasmids used in this study were constructed using the pGPU6/GFP/Neo plasmid (GenePharma, Shanghai, China). The targeting sequences were 5′-GCA TCA CGC TTG ACC CAT T-3′ (sh-IGFL2-AS1) for the human IGFL2-AS1 transcript and 5′-TTC TCC GAA CGT GTC ACG T-3′ (sh-NC) for the non-targeting control [8]. Plasmids were transiently transfected using Lipofectamine 2000 (Invitrogen, Carlsbad, CA, USA) according to the manufacturer’s instructions, after which stably expressing cells were selected by culturing in the presence of the neomycin analog, G418. Cells were incubated for 14 days, with the replacement of G418-containing medium every 3 days. After 2 weeks, green fluorescent protein (GFP)-positive clones were isolated and maintained in the presence of G418. Fluorescence images were obtained using an Eclipse Ti fluorescence microscope (Nikon, Tokyo, Japan).

### 4.7. Measurement of Cell Viability

Cell viability was assessed by MTT [3-(4,5-dimethylthiazol-2-yl)-2,5- diphenyltetrazolium bromide] assay. Briefly, cells were seeded at a density of 5 × 10^4^ cells per well of a 6-well plate and allowed to adhere at 37 °C in a humidified 5% CO_2_ environment. For γ-irradiation, cells were exposed to a single dose of 0, 3, 6, or 9 Gy using a Biobeam 8000 (Gamma-Service Medical GmbH, Leipzig, Germany) with a Cs-137 source. After culturing cells for 3 days, 10 μL MTT solution (5 mg/mL; Amresco, Solon, OH, USA) was added, and cells were incubated for an additional 4 h. The medium was then carefully removed and, after washing cells with phosphate-buffered saline (PBS), 200 μL dimethylsulfoxide (DMSO) was added and the optical density of each well was measured at 570 nm with a microplate reader. The reported results are the average of triplicate samples.

### 4.8. Measurement of Cell Proliferation

For measurement of cell proliferation, stably transfected cells were seeded at a density of 1 × 10^5^ cells per well of a 12-well plate and allowed to adhere at 37 °C in a 5% CO_2_ incubator. For γ-irradiation, cells were exposed to a single 3-Gy dose (1.3 Gy/min) using a Biobeam 8000 (Gamma-Service Medical GmbH) with a Cs-137 source. The plate was then placed into an automated live cell imaging system (Celloger Mini Plus; CURIOSIS, Seoul, Republic of Korea) and each well was imaged every 2 h for 48 h at 37 °C and 5% CO_2_. The degree of cell confluence in each image was calculated using Celloger Mini Plus analysis software (CURIOSIS).

### 4.9. Wound-Healing Assay

The wound-healing process was assessed by first seeding stably transfected cells at a density of 5 × 10^5^ cells per well in a 12-well plate and incubating at 37 °C and 5% CO_2_ until cells formed a monolayer (100% confluence). The cell monolayer was then scratched in a straight line using a sterile pipette tip, mimicking an incision wound. After removing floating cells by washing twice with PBS, adherent cells were exposed to a single 3-Gy dose of γ-irradiation (1.3 Gy/min) using a Biobeam 8000 with a Cs-137 source. The plate was placed in a Celloger Mini Plus automated live cell imaging system (CURIOSIS), and the scratched region was monitored every 2 h for 48 h at 37 °C and 5% CO_2_. Images were analyzed using Celloger Mini Plus analysis software (CURIOSIS).

### 4.10. Clonogenic Assay

A clonogenic assay was performed as described in the previous paper [37]. Cells were seeded at various densities (300–600 cells per well) in 6-well plates and incubated at 37 °C and 5% CO_2_ until cells adhered. Then, cells were exposed to a single dose of 0, 1.5 and 3 Gy of γ-irradiation (1.3 Gy/min) using a Biobeam 8000 with a Cs-137 source. Cells were maintained at 37 °C and 5% CO_2_ for 14 days, and then cells were fixed with 4% paraformaldehyde at 4 °C for 20 min and stained with 0.5% crystal violet at RT for 2 h. A colony was considered viable if it contained >50 cells. Colonies were counted with the ImageJ version 1.53 (National Institutes of Health, MD, USA) and the results are expressed as surviving fractions.

### 4.11. Western Blot Analysis

Cells were lysed with lysis buffer (Cell Signaling Technology, Danvers, MA, USA), and equal amounts of protein were resolved by sodium dodecyl sulfate-polyacrylamide gel electrophoresis (SDS-PAGE) on 8–12% gels and transferred to nitrocellulose membranes. The membranes were blocked by incubating with 5% non-fat dry milk in PBS containing 0.2% Tween-20 (PBST) for 1 h, and then incubated with primary antibodies overnight at 4 °C. After three washes with PBST, blots were incubated with horseradish peroxidase (HRP)-conjugated secondary antibodies against mouse (#A120-101P) or rabbit (#A90-116P) (Bethyl Laboratories, Inc., Montgomery, TX, USA) for 1 h at room temperature. Protein bands were detected using enhanced chemiluminescence (ECL) reagents (Amersham Pharmacia Biotechnology, Amersham, UK) and X ray film (AGFA, Mortsel, Belgium). Primary antibodies against ARPP19 (#MBS9609642) were obtained from MyBioSource (San Diego, CA, USA); phospho-AKT (#4060) and AKT (#4691) were obtained from Cell Signaling Technology; c-Myc (#sc-764), cyclin D1 (#sc-753), E-cadherin (#sc-8426), and β-actin (#sc-47778) were purchased from Santa Cruz Biotechnology (Santa Cruz, CA, USA).

### 4.12. Statistical Analysis and Graphical Representation

Statistical analyses were performed using GraphPad Prism 5 or Microsoft Excel 2010. Data obtained from multiple independent experiments are presented as means ± standard deviation (SD). The statistical significance of differences was analyzed by Student’s t-test or one-way ANOVA with Tukey post-hoc test. *p*-value < 0.05 was considered significant.

## Figures and Tables

**Table 1:** Relationship between IGFL2-AS1 expression level and prognosis of different types of cancer in the Kaplan-Meier Plotter.

Cancer Type	Survival (Months)	
	Low Expression Cohort	High Expression Cohort
Bladder carcinoma *	55.67	31.37
Breast cancer **	87.87	76.53
Esophageal squamous cell carcinoma *	42.10	22.7
Head-neck squamous cell carcinoma *	61.27	42.97
Kidney renal clear cell carcinoma **	53.00	28.87
Kidney renal papillary cell carcinoma **	86.97	51.90
Lung adenocarcinoma *	55.10	45.97
Lung squamous cell carcinoma *	63.73	49.00
Ovarian cancer *	48.20	44.70
Pancreatic ductal adenocarcinoma *	24.40	17.73
Sarcoma *	77.47	64.70
Uterine corpus endometrial carcinoma *	108.37	47.60

* Median survival and ** Upper quartile survival.

## Data Availability

Data are contained within the article and Supplementary Material.

## Supplementary Materials

The supporting information can be downloaded at: https://www.mdpi.com/article/10.3390/ijms24020978/s1.
