# Supplementary material for: IGFL2-AS1, a Long Non-Coding RNA, Is Associated with Radioresistance in Colorectal Cancer
Source: Int J Mol Sci. 2023 Jan 4;24(2):978. doi: 10.3390/ijms24020978 (PMC9866146; doi:10.3390/ijms24020978)
Supplement: Supplementary file 1 [file ijms-24-00978-s001.zip › Supplementary figures.pdf]

Supplementary figures

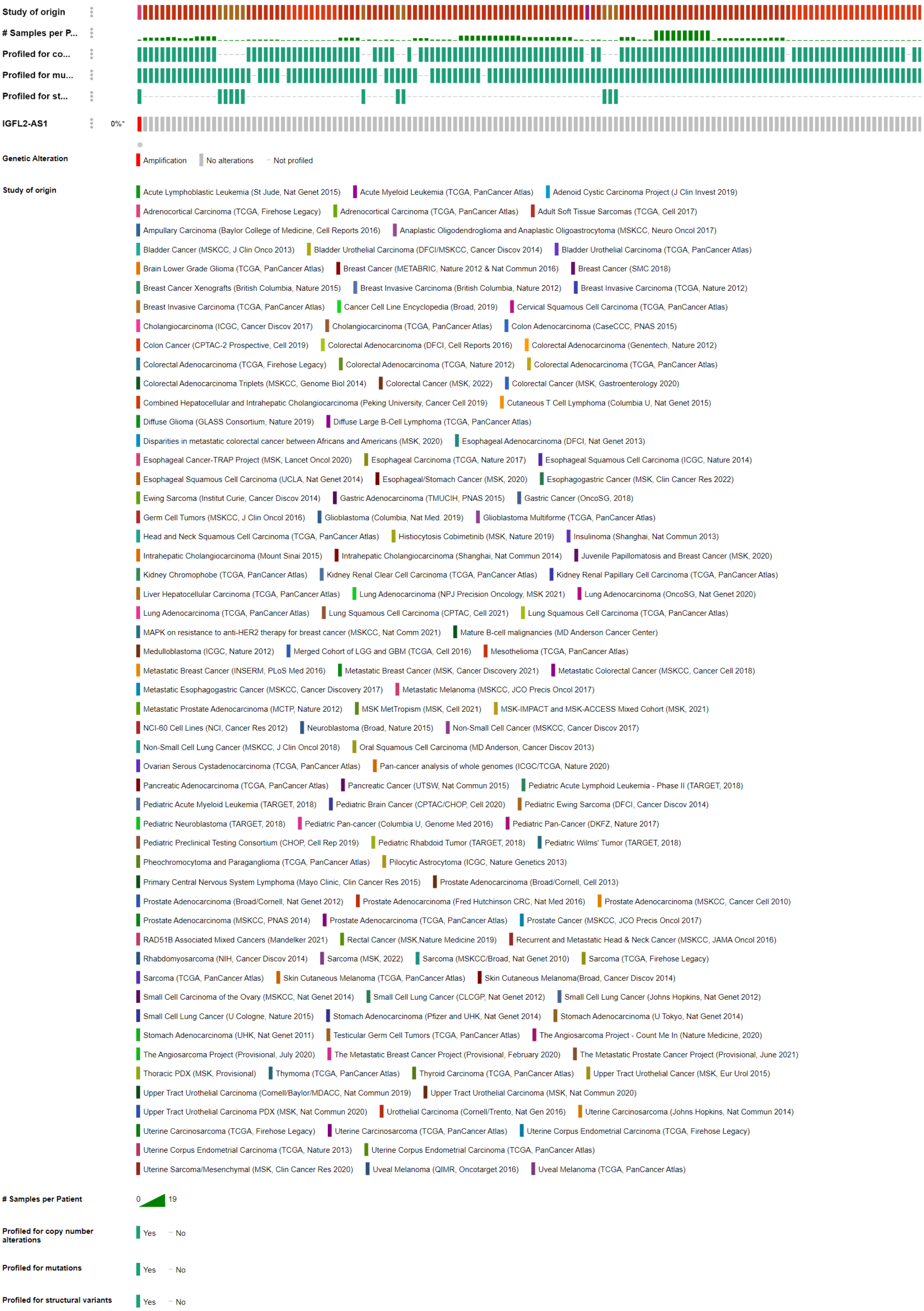

Figure S1. Candidate gene validation and genetic alterations in cancers. The candidate gene was queried for genetic alterations across 153 different cancer studies (http://cbioportal.org/ accessed December 20, 2022). Genetic alterations are depicted graphically and color coded: amplification, red; no alterations, gray.

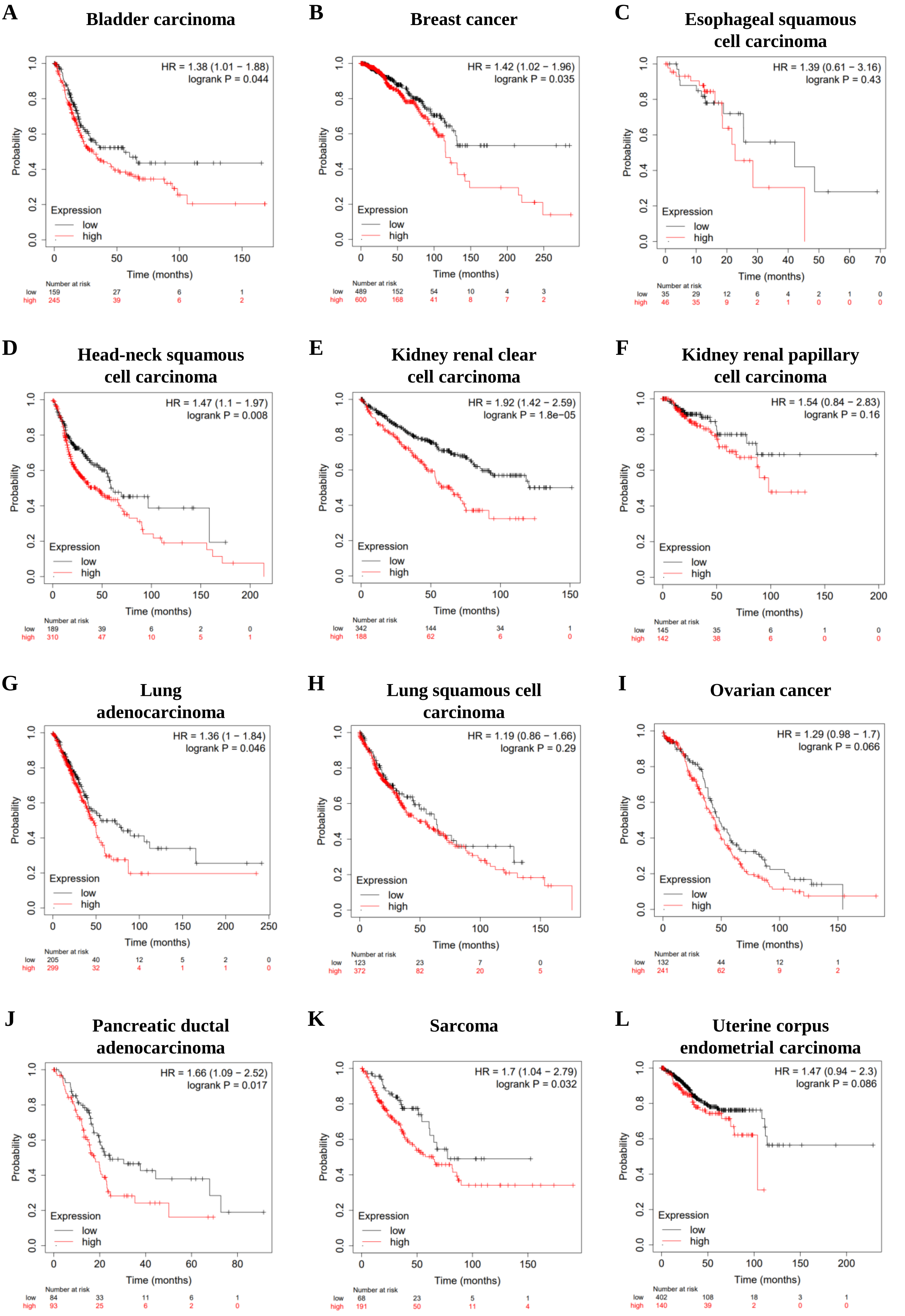

Figure S2. Overall survival (OS) analysis of IGFL2-AS1 genes. OS analysis of IGFL2-AS1 genes was performed using the Kaplan-Meier method with a log-rank statistical test. The patients were categorized into a high-expression group (red) and a low-expression group (black) according to the median or upper quartile expression level of IGFL2-AS1 as indicated. High expression groups exhibited significant associations with poor OS time in patients with (**A**) bladder carcinoma, (**B**) breast cancer, (**C**) esophageal squamous cell carcinoma, (**D**) head-neck squamous cell carcinoma, (**E**) kidney renal clear cell carcinoma, (**F**) kidney renal papillary cell carcinoma, (**G**) lung adenocarcinoma, (**H**) lung squamous cell carcinoma, (**I**) ovarian cancer, (**J**) pancreatic ductal adenocarcinoma, (**K**) sarcoma, and (**L**) uterine corpus endometrial carcinoma.

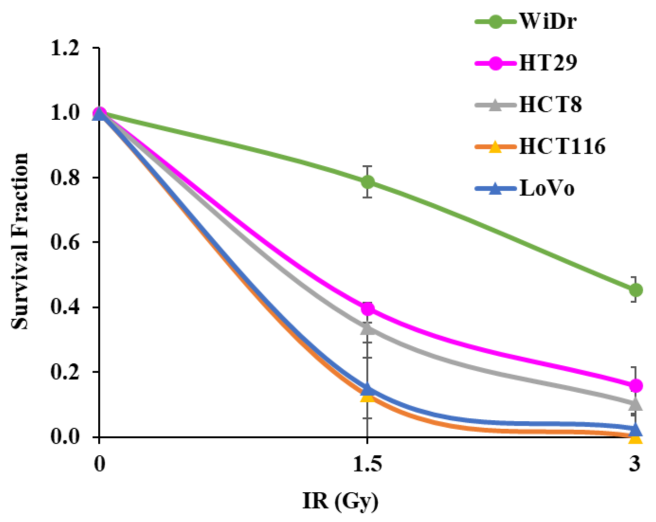

Figure S3. Clonogenic assay of CRC cell lines after irradiating with doses of 0, 1.5 and 3 Gy (n = 3 independent experiments). Colonies were obtained on the 14th day after irradiation and the results are expressed as surviving fraction. Data are normalized to those of non-irradiated controls and are presented as means ± SD.

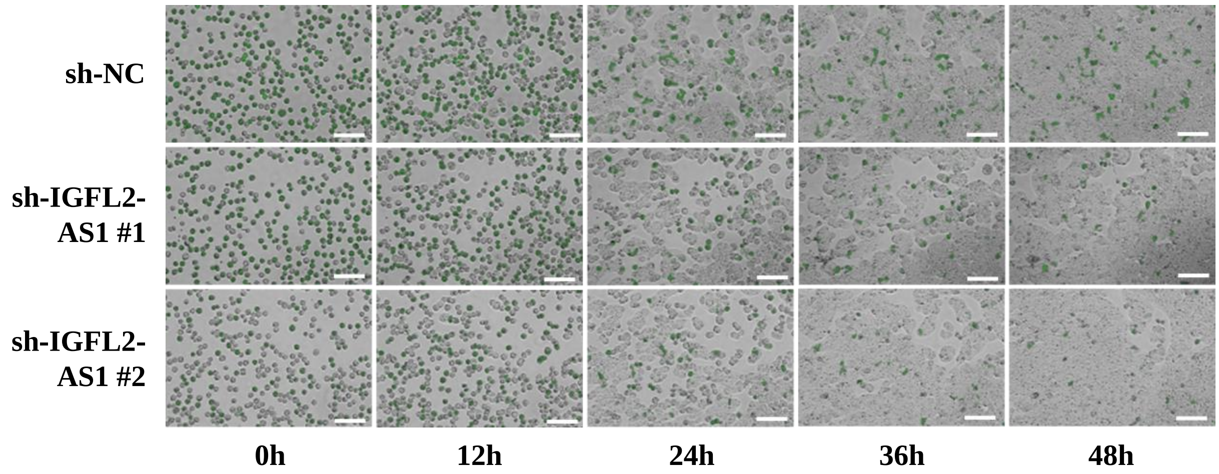

Figure S4. Time-lapse images of IGFL2-AS1–transfected CRC cell lines. Proliferation of the indicated cell lines was measured using a Celloger Mini Plus time-lapse microscopy system. Representative time-lapse images of the indicated cell lines at 0, 12, 24, 36, and 48 hours are shown. Scale bars, 200 μm.

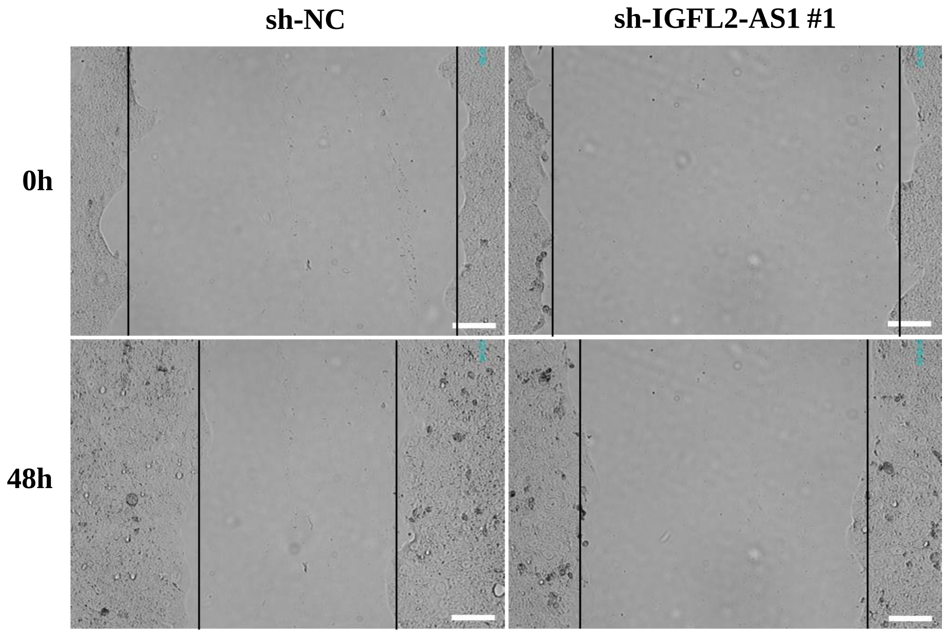

Figure S5. Images of wound healing in IGFL2-AS1–transfected CRC cell lines. Wound-healing assays in the indicated cell lines, performed using a Celloger Mini Plus time-lapse microscopy system. Representative time-lapse images of wound-healing assays obtained over a 48-h period after scratching (at 0 hour) are shown. Scale bars, 200 μm.

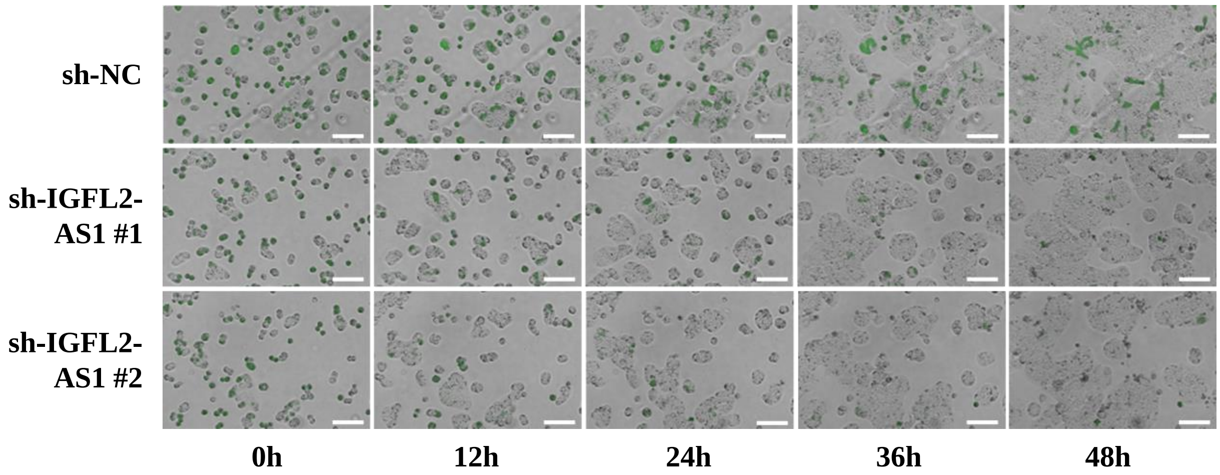

Figure S6. Time-lapse images of IGFL2-AS1–transfected CRC cells following irradiation. Proliferation of the indicated cell lines after irradiation at a dose of 3 Gy, measured using a Celloger Mini Plus time-lapse microscopy system. Representative time-lapse images of the indicated cell lines at 0, 12, 24, 36, and 48 hours are shown. Scale bars, 200 μm.

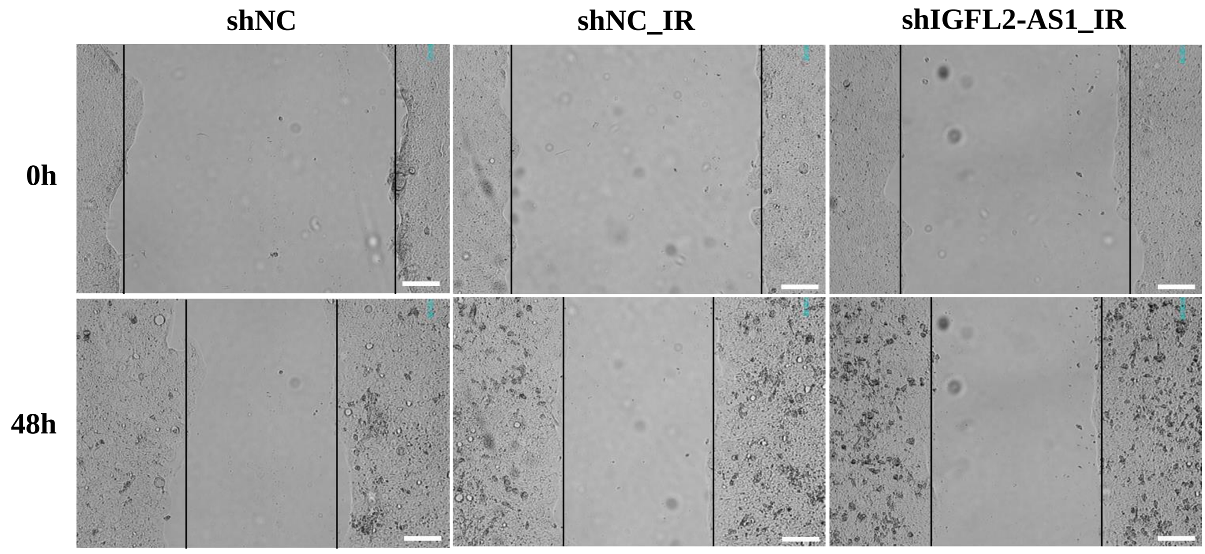

Figure S7. Images of wound healing in IGFL2-AS1–transfected CRC cells following irradiation. Wound-healing assays of the indicated cell lines after irradiation at a dose of 3 Gy were performed using a Celloger Mini Plus time-lapse microscopy system. Representative time-lapse images of wound-healing assays obtained over a 48 hours period after scratching (at 0 hour) are shown. Scale bars, 200 μm.

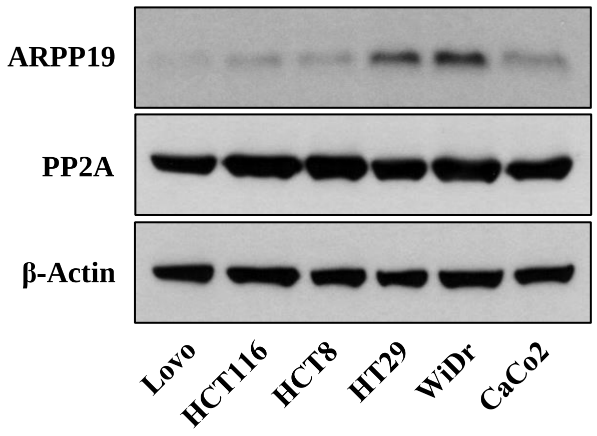

Figure S8. Western blot analysis of ARPP19 and PP2A expression in CRC cell lines. β-actin was used as a loading control.
