# Supplementary material for: IGFL2-AS1, a Long Non-Coding RNA, Is Associated with Radioresistance in Colorectal Cancer
Source: Int J Mol Sci. 2023 Jan 4;24(2):978. doi: 10.3390/ijms24020978 (PMC9866146; doi:10.3390/ijms24020978)
Supplement: Supplementary file 1 [file ijms-24-00978-s001.zip › Table S1 Patients (Final).pdf]

Table S1. Association between IGFL2-AS1 and clinical features

Sample No.	RR-1	RR-2	RR-3	RS-1	RS-2	RS-3
**Sex**	M	M	F	M	M	F
**Age (years)**	77	71	76	49	64	62
**Clinical stage**	T3N+	T3N+	T3N+	T3N+	T2N+	T3N+
**Regression**	Minimal	Minimal	Minimal	Complete	Complete	Complete
**ypStage**	T3N1M0	T3N1M0	T3N0M0	T0N0M0	T0N0M0	T0N0M0
**IGFL2-AS1 Expression**	4.2	0.5	3.4	0.3	0.3	0.2

Abbreviation: ypStage, pathologic stage after neoadjuvant treatment at surgical specimen.
